# Oligodendrocytes in Schizophrenia

**DOI:** 10.1155/2011/249768

**Published:** 2011-09-18

**Authors:** Haiyun Xu, Vahram Haroutunian, George Bartzokis, Martha E. Shenton

**Affiliations:** ^1^Department of Anatomy, Southern Illinois University Carbondale, Carbondale, IL 62901, USA; ^2^Department of Psychiatry, Mount Sinai School of Medicine, NY 10128, USA; ^3^Department of Psychiatry and Biobehavioral Sciences, The David Geffen School of Medicine at UCLA, Los Angeles, CA 90095 6968, USA; ^4^Department of Psychiatry and Radiology, Brigham and Women's Hospital, Harvard Medical School, Boston, MA, USA; ^5^The Clinical Neuroscience Division, Laboratory of Neuroscience, VA Boston Healthcare System, Harvard Medical School, Boston, MA, USA

Despite the many neuroimaging studies that suggest gray matter volume reductions in schizophrenia, there is no compelling postmortem evidence to suggest neuronal loss, nor is there a distinctive or specific signature of gray matter abnormalities in schizophrenia. Instead, there appears to be no observed focal lesion that characterizes gray matter pathology in schizophrenia. This state of affairs has led to a movement away from conceptualizing schizophrenia as resulting from a single lesion, to conceptualizing schizophrenia as arising from abnormal communication between brain regions. Abnormal connectivity between brain regions is not a new concept, as Bleuler (1911) postulated such abnormalities. What is new, however, is an appreciation that brain regions that are not spatially proximal may be connected functionally into neural networks and that to understand altered neural connectivity we need to have a better understanding of the connections between brain regions which are made possible by white matter, the main infrastructure in the brain that makes possible long distance communication among neurons. Accordingly, a focus on white matter connections in the brain has become increasingly of interest in schizophrenia, particularly in the areas of imaging studies, postmortem studies, and new animal models of schizophrenia. This special issue focuses on schizophrenia and oligodendrocytes, the latter a class of neuroglia that give rise to myelin. The primary aim of this special issue is to understand and to clarify further white matter pathology in schizophrenia and how it may contribute to disconnectivity among brain regions, which results in the observed cognitive, behavioral, and clinical symptoms in this disorder.

The first paper begins with a description of the theory that schizophrenia reflects a disorder of connectivity. The proposition put forth is that schizophrenia is, at least in part, the result of abnormal communication between brain regions that may be spatially distant but nonetheless functionally connected. As white matter is the main infrastructure for brain connectivity, this paper focuses on the use of a magnetic resonance imaging (MRI) technique called diffusion tensor imaging (DTI) to gain insight into the role of white matter abnormalities in schizophrenia. This paper also focuses on the functional implications of white matter abnormalities, particularly with regard to the possible role of myelin in modulating the transmission velocity of neural discharges. The paper ends with a speculative hypothesis about the relationship between gray matter and white matter abnormalities in schizophrenia.

The second paper also focuses on altered neuronal connectivity. Here, however, the focus is on altered connectivity and impaired myelination in a postmortem study of schizophrenia and normal controls, where electron microscopy is used to study myelinated fibers and oligodendrocytes. This morphometric study examines myelinated fibers in the prefrontal cortex in both gray and white matter. Six types of abnormal fibers and ultrastructural alterations are described in the schizophrenia sample. The study reveals increased pathological fibers in gray matter in both young and elderly patients that are associated with positive symptoms, while in elderly patients, the frequency of pathological fibers in white matter is increased and these changes are associated with more negative symptoms. The key implication here is that altered myelinated fibers in white matter in schizophrenia likely follow alterations of myelinated fibers in gray matter that occur earlier in the course of illness.

The third paper is a postmortem study on age-related changes in the number of oligodendrocytes in prefrontal cortex in schizophrenia, bipolar disorder and major depression. The study reveals an age-related increase in numerical density of oligodendrocytes in layer VI and adjacent white matter of BA9 and 10 that is seen only in normal controls and not in schizophrenia or in mood disorders. The absence of the normal age-related increase in oligodendrocytes in patients suggests that this aspect of normal brain development is dysregulated in schizophrenia and in mood disorders, and confirms postmortem and imaging data, further highlighting the fact that the absence of a normal age-related increase in oligodendrocytes is a key shared feature in these psychiatric populations.

The fourth paper reviews white matter abnormalities in schizophrenia, including imaging and postmortem findings on the disconnectivity theory of schizophrenia, a major theme that underlies the three previous papers in this special issue. This paper then reviews some of the white matter diseases that commonly lead to psychotic symptoms and then reviews transgenic and mutant mouse models of schizophrenia. More specifically, genetic mouse models such as Plp1 transgenic mice and mutant mice heterogeneous for either NRG1 or its receptor erbB4, as well as Nogo-A- deficient mice are reviewed. This is followed by a focus on the myelin toxicity model of mice fed cuprizone. In the early stage (the second and third week) of the cuprizone feeding, mice show higher dopamine levels and lower norepinephrine levels in their prefrontal cortex, along with behavioral changes indicative of increased CNS activity. In the late stage (weeks 4–6), when demyelination and oligodendrocyte loss are obvious, mice display cognitive deficits as well as deficits in social interactions that are reminiscent of social withdrawal seen in patients with schizophrenia. Importantly, this cuprizone-feeding mouse may be used as an in vivo platform for psychopharmacological studies testing the effects of antipsychotic drugs on white matter alterations and associated behavioral changes, in addition to providing a new animal model of schizophrenia.

The fifth and final paper of this special issue focuses on abnormal behavior and microstructural changes in juvenile mice that are repeatedly exposed to nonneurotoxic levels of amphetamine. These mice are compared with nonamphetamine treated mice. Oligodendrocyte numbers and three proteins expressed in mature oligodendrocytes are investigated in these young male mice at sacrifice. Treated mice showed higher locomotion and impaired spatial working memory, along with lowered Noga-A and GST-pi proteins, and lower MBP proteins, in frontal cortex and hippocampus and fewer mature oligodendrocytes in frontal cortex and corpus callosum, as well as lower MBP staining in frontal cortex, corpus callosum, and hippocampus, than untreated mice. These differences suggest that in wild-type mice late developing white matter is vulnerable to amphetamine and may lead to compromised white matter with increased dopamine in specific brain regions. Not only do these findings explain well the increased locomotion and impaired spatial working memory observed in amphetamine-treated mice, but also may serve as a principle for the amphetamine-treated mouse being used as a novel animal model of schizophrenia.

To summarize, compared to the focus on gray matter and neurons, research on white matter and myelin/glial has been sparse in schizophrenia. In this special issue, neuroimaging findings are presented that suggest that myelin abnormalities in schizophrenia may underlie the white matter abnormalities observed using diffusion tensor imaging techniques. Data from postmortem studies also confirm the presence of oligodendrocyte and myelin abnormalities in schizophrenia as well as mood disorders. Oligodendrocytes provide protection and improve communication between brain regions and the oligodendroglia-producing myelin is approaching maturity in the same time frame as the onset of symptoms. Further, normal age-related white matter increases and related gray matter changes that likely predate the white matter changes are dysregulated in schizophrenia and may contribute to the clinical symptomatology of the disease. Finally, the recently developed animal models are not only of help in understanding connectivity abnormalities in schizophrenia but also provide novel platforms to test novel therapeutic approaches for schizophrenic patients. Treatments specifically targeting white matter, for example, may improve on the efficacy of those treatments currently in use.

*Martha E. Shenton* *Martha E. Shenton* *Haiyun Xu* *Haiyun Xu* *Vahram Haroutunian* *Vahram Haroutunian* *George Bartzokis* *George Bartzokis*

